# Validation of the Epi2SensA Method Using the EpiDerm™ Model for Skin Sensitization Testing Under OECD TG442D

**DOI:** 10.3390/toxics14040295

**Published:** 2026-03-28

**Authors:** Christian Pellevoisin, Hajime Kojima, Sebastian Hoffmann, Takao Ashikaga, Timothy Landry, Celina Romero, Kalyani Guntur, Mitchell Klausner, Jennifer Stadnicki, Helge Gehrke, Robert Mills-Goodlet, Niki Panousi, Victor J. Johnson, Gary R. Burleson, Kazuto Narita, Shigehiro Tachibana, Kohichi Kojima, Jan Markus, Alexander Armento

**Affiliations:** 1 Mattek Corporation Now Part of Sartorius, Ashland, MA 01721, USA; 2 Urbilateria, 37540 Tours, France; 3 Department of Pharmaceutical Engineering, Faculty of Engineering, Sanyo-Onoda City University, Yamaguchi 756-0884, Japan; 4 National Institute of Health Sciences, Kawasaki 201-0821, Japan; 5 Seh Consulting + Services, 33106 Paderborn, Germany; 6 Eurofins Medical Device Testing Munich GmbH, D-82152 Planegg, Germany; 7 Burleson Research Technologies, Inc., Morrisville, NC 27560, USA; 8 Food and Drug Safety Center, Hadano 729-5, Japan; 9 Mattek In Vitro Life Science Laboratories, Now Part of Sartorius, 821 05 Bratislava, Slovakia

**Keywords:** Epi2SensA, skin sensitization, reconstructed human epidermis, interlaboratory validation, OECD TG 442D, alternative testing methods, in vitro assay, keratinocyte activation

## Abstract

The Epi2SensA method is a method similar to the validated EpiSensA assay for assessing the skin sensitization potential of chemicals. The Epi2SensA protocol includes adaptation (changes to exposure conditions and the controls) for using an alternative reconstructed human epidermis (RhE) model, the EpiDerm™ model. The interlaboratory validation study evaluated the reliability and predictive capacity of Epi2SensA according to OECD Performance Standards. Four laboratories (Mattek, Now Part of Sartorius, Eurofins Munich, Burleson Research Technologies, Inc., and Food and Drug Safety Center) conducted blinded testing of 20 coded reference substances representing various chemical categories and sensitization potencies. Statistical analysis using modified acceptance criteria (a 60% cell viability threshold) and a modified prediction model (requiring at least two positive gene markers) demonstrated substantially improved performance compared to the original EpiSensA criteria. The between-laboratory reproducibility (BLR) was 85%, the average within-laboratory reproducibility (WLR) was 83.3%, and the average predictivity parameters were 88.1% for sensitivity, 88.9% for specificity, and 88.3% for accuracy. Epi2SensA achieved performance metrics comparable to the validated reference method (EpiSensA), supporting regulatory acceptance of the Epi2SensA assay using the EpiDerm™ model (Mattek Corporation, Now Part of Sartorius, Ashland, MA, USA) as an alternative RhE source for OECD TG 442D skin sensitization testing.

## 1. Introduction

Since the publication of the “The Adverse Outcome Pathway for Skin Sensitization Initiated by Covalent Binding to Proteins” in 2012 [1], several methods based on the modeling of Adverse Outcome Pathway (AOP) Key events (KE) have been validated and integrated into TG 442. These methods include DPRA, ADRA, kDPRA [2] for KE1 (protein binding, TG 442C), KeratinoSens™, LuSens, and EpiSensA [3] for KE2 (keratinocyte activation, TG 442D), and h-CLAT, U-SENS™, IL-8 Luc, GARDskin [4] for KE3 (dendritic cell activation, TG 442E). These methods illustrate the paradigm shift toward mechanistically based safety assessments to replace methods that are historically animal-based. Currently, no single assay is accepted to capture the complex, multi-step sensitization process. Comprehensive hazard evaluation requires integration of test batteries within Integrated Approaches to Testing and Assessment (IATA). The OECD Guideline 497 establishes Defined Approaches (DAs) that combine multiple in silico, in chemico, and in vitro information sources through fixed data interpretation procedures, for hazard identification and GHS potency sub-categorization without expert judgment [5]. DAs such as the “2 out of 3” approach and Integrated Testing Strategy (ITS) provide structured frameworks for combining KE1, KE2, and/or KE3 data, enhancing predictive capacity and regulatory consistency while eliminating reliance on animal testing. Most validated methods use 2D cell lines in aqueous systems, limiting applicability to hydrophobic chemicals or those which can form a stable suspension. Limited metabolic capacity in immortalized cell lines can also compromise detection of pro-haptens requiring metabolic activation, increasing the risk of false negatives. In 2024, the RhE-based sensitization assay (EpiSensA), an in vitro test method addressing KE2, was validated and incorporated into OECD TG 442D. In this method, gene expression of four mechanistically relevant markers reflecting the keratinocyte response to the early phase of skin sensitization is measured after exposure of a chemical to the RhE model [6]. The validation of this method demonstrates that the use of an RhE model as an experimental system enables direct testing of hydrophobic substances and effectively detects pre/pro-haptens [7]. The air–liquid interface culture approach with direct topical exposure to the tissue makes RhE models particularly suitable for testing hydrophobic substances, complex mixtures, and formulations that are not readily compatible with aqueous systems [8]. The EpiSensA test method was originally developed in 2013 with the Mattek EpiDerm RhE model [9]. It was then optimized [10] and validated in 2023 with the LabCyte EPI-MODEL24 (J-TEC, Gamagori, 443-0022, Japan), mainly available in Japan. Epi2SensA is a method with an adapted protocol for an alternative RhE model, the EpiDerm™ (EPI-200, Mattek Corporation, Now Part of Sartorius, Ashland, MA, USA). The purpose of this study was to validate Epi2SensA as a similar method to EpiSensA. The interlaboratory validation was conducted under blind conditions according to OECD Guidance Document No. 34 and the Performance Standards for EpiSensA-like methods published by OECD in 2024 [11]. The validation assessed both the within-laboratory reproducibility (WLR) and between-laboratory reproducibility (BLR), as well as the predictive capacity (accuracy, sensitivity, and specificity) of Epi2SensA compared to in vivo skin sensitization classifications. Like EpiSensA, Epi2SensA aims to be integrated into OECD TG442D as part of an Integrated Approach to Testing and Assessment (IATA) within the AOP framework to assess sensitization potential for hazard classification and labeling. Integration of Epi2SensA into OECD TG442D will provide laboratories with multiple RhE supplier options and ensure reliable worldwide supply chain accessibility.

## 2. Materials and Methods

### 2.1. Study Design and Organization

The validation study followed a two-phase design conducted between June 2024 and August 2025. Phase 1 involved method transfer and training of three naïve testing laboratories. The naïve laboratories were provided with a Standard Operating Procedure (SOP), which included detailed instructions for handling the RhE and how to conduct the assay. For the transfer of the method, a set of four compounds (glycerol, eugenol, salicylic acid, and 2-aminophenol) for which VRM data were available but not belonging to the validation set were arbitrarily selected. Solvent selection and solubility, dose range-finding viability experiment, and the main study of the Epi2SensA testing protocol were performed by each laboratory. After the assay was successfully transferred to all laboratories, Phase 2 comprised blind testing of coded reference substances to assess assay reproducibility and predictive performance. An interim analysis was conducted after initial rounds of testing to assess whether reproducibility parameters remained within acceptable ranges before proceeding to complete testing of all reference substances.

**Figure 1:**
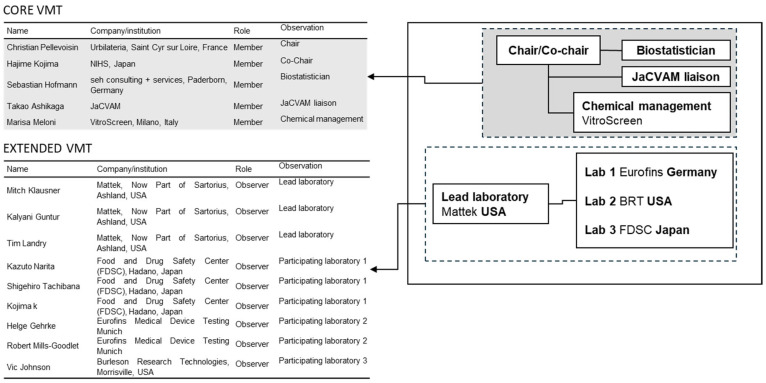
Schema of the organization of the validation with the composition of the core and extended Validation Management Team (VMT) and the list of participating laboratories.

The study was organized by a Validation Management Team (VMT) comprising five core members and nine observers from participating laboratories (Figure 1). The VMT supervised all aspects of the study including study planning, laboratory training, chemical coding and distribution, data collection, statistical analysis, and report preparation. This structure ensured adherence to OECD principles while maintaining scientific oversight and data integrity.

### 2.2. Participating Laboratories

Four laboratories participated in this validation study: Mattek Corporation, Now Part of Sartorius (Ashland, MA, USA—lead laboratory), Burleson Research Technologies, Inc. (BRT; Morrisville, NC, USA), Eurofins Medical Devices Testing (Munich, Germany) and Food and Drug Safety Center (Hadano, Japan). The lead laboratory (Mattek) was responsible for final Standard Operating Procedure (SOP) development and training of participating laboratories. While FDSC initially committed to testing the complete reference substance set, resource limitations necessitated restriction to a subset of eight substances for between-laboratory reproducibility assessment, with testing conducted once per substance. The three main laboratories (Mattek, Eurofins, BRT) tested the complete reference set of 20 substances: 12 tested in triplicate for WLR assessment and 8 tested once for BLR assessment, representing a total of 44 coded samples per laboratory (see Supplementary Table S1).

### 2.3. Test Chemicals

The validation set included 20 reference substances (Table 1) recommended by the OECD Performance Standards (PS) for validation of similar EpiSensA methods [11]. This set of reference substances covers the full range of in vivo skin sensitization effects as established by the Local Lymph Node Assay (LLNA)EC3 value (non-sensitizer, weak, moderate, strong and extreme). Reference substances include 14 sensitizers (UN GHS Categories 1A or 1B) and 6 non-sensitizers with in vivo predictions derived from LLNA data.

**Figure 2:**
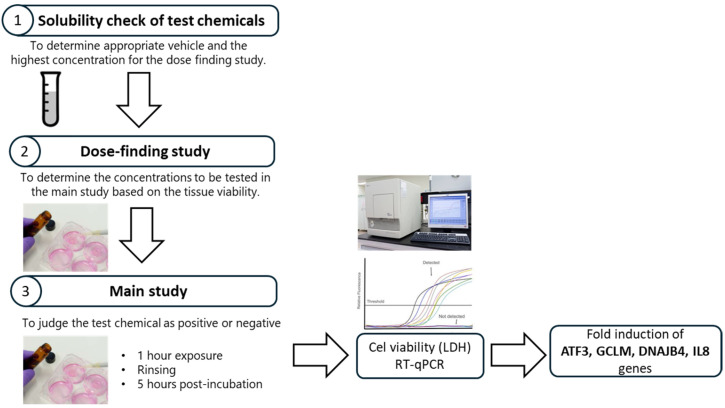
Different steps of the Epi2SensA method. For a given chemical, the first step is to identify the maximum concentration (solubility) in the appropriate solvent. Appropriate doses for the main study are determined in the dose finding which measures cytotoxicity as a function of test article concentration, using the LDH assay. In the main study, the expression of the 4 genes involved in skin sensitization are measured by RT-qPCR.

Chemical acquisition, coding, and distribution were subcontracted to VitroScreen (Milan, 20149, Italy). For practical feasibility and tissue consumption, the VMT implemented a grouping strategy for the dose finding study of the Epi2SensA protocol (Figure 2). Ten coded samples underwent full concentration-finding studies by each laboratory, while the remaining 34 coded samples were assigned to three predefined concentration ranges based on historical Mattek cytotoxicity data. This strategy enabled reproducibility of concentration finding between laboratories, which substantially reduced tissue requirements since it eliminated the need for individual concentration-finding studies for all 44 coded substances, while maintaining blind conditions.

### 2.4. Reconstructed Human Epidermis Model: EpiDermTM

The EpiDerm™ model (EPI-200, from Mattek Corporation, Now Part of Sartorius, Ashland, MA, USA and Mattek In Vitro Life Science Laboratories, Now Part of Sartorius, Bratislava, Slovakia) was utilized throughout this validation study. The EpiDerm three-dimensional RhE model consists of normal human keratinocytes cultured on porous membrane inserts at the air–liquid interface to form a multilayered, highly differentiated epidermis with morphological characteristics closely resembling native human skin [12].

### 2.5. Chemical Solubility Assessment

For each test chemical, solubility was assessed in three vehicle options in the following order of preference: (1) acetone:olive oil (AOO; 4:1, 20% *v/v* olive oil in acetone); (2) distilled water (DW); (3) 50% ethanol in DW (50% EtOH). If a chemical was not soluble or did not form a stable dispersion in these vehicles, serial dilutions (2-fold) beginning at 50% down to 0.0122% were performed to identify the highest soluble concentration in each solvent. For such test substance, the appropriate solvent was selected as the one producing the highest soluble concentration (or stable suspension).

### 2.6. Dose-Finding Study

Duplicate EpiDerm™ tissues were exposed to 10 μL of test chemical using up to six concentrations per test chemical to identify concentrations eliciting excessive cytotoxicity. Following 1 h exposure, the RhEs were rinsed 15 times with 0.5mL of DPBS and incubated in fresh medium post-rinse for 5 h. For valid runs, results of at least one concentration with ≥80% mean tissue viability were required; this threshold was subsequently modified to ≥60% based on analysis of the assay performance data. Tissue viability was determined by the release of LDH (Lactate Dehydrogenase) into the culture medium, as measured using an LDH assay kit (Sigma, 11644793001) according to the manufacturer’s instructions. LDH is a stable cytoplasmic enzyme which is rapidly released into the tissue culture medium upon damage to the plasma membrane. Tissue viability was calculated using the equation below where Δabs represents the absorbance measured at 490 nm minus the absorbance at reference wavelength (≥650 nm) and killed control representing the maximum of LDH released by tissues exposed to 50 μL of 10% Triton-X-100 that were added into the basolateral medium:

$$cell\;viability\;\left(\%\right)=100-\;\frac{\Delta abs.\;of\;test\;chemical\;treatment-\Delta abs.\;of\;vehicle\;control)}{mean\;\Delta abs.\;of\;killed\;control-\Delta abs.\;of\;vehicle\;control}*100$$

For the killed control tissues, the protocol was optimized for the EpiDerm^TM^ model by comparing different conditions to get the maximum LDH release. The viability determined by the LDH assay was compared to tissue viability measured using the 3-[4,5-dimethylthiazol-2-yl]-2,5 diphenyl tetrazolium bromide (MTT) assay.

### 2.7. Main Study

The main study evaluated gene expression changes following tissue exposure to test substances. Tissues in triplicates were exposed to 10 μL of test substance (typically four concentrations per chemical), appropriate vehicle controls, and the two positive controls, Clotrimazole 1.56% and 4-Nitrobenzyl bromide (4-NBB) 0.78%. After the 1 h exposure, the RhE tissues were rinsed 15 times with 0.5 mL of DPBS and post-incubated for 5 h. Then, tissues were harvested, lysed with RNA lysis buffer, and frozen at −80°C until being processed for total RNA extraction using the RNAqueous Total RNA Isolation Kit (ThermoFisher Scientific). The isolated RNA was used to synthesize cDNA using the RT2 First Strand Kit (Qiagen). Quantitative reverse transcription–polymerase chain reaction (RT-qPCR) was conducted to measure fold induction of four marker genes (ATF3, GCLM, DNAJB4, IL-8) relative to the vehicle control.

A run was considered valid if the following acceptance criteria were met:

- The tissue viability of at least two of the tissues exposed to the vehicle control was ≥95%.
- The mean tissue viability of both positive controls was ≥80%.
- For the positive control 1.56 *w*/*v*% clotrimazole, the mean values of fold induction for ATF3 and IL-8 exceeded the cut-off value.
- For the positive control 0.10 w/v% 4NBB, the mean values of fold induction for GCLM and DNAJB4 exceeded the cut-off value.

If these requirements were not met, the run was considered invalid and was repeated. For a specific concentration of test substance to be accepted, the cell viability must have been ≥80% using the validated reference method (VRM) criteria or ≥60% using Epi2SensA optimized acceptance criteria, and the mean GAPDH Ct value must have been within ±1 of the mean GAPDH Ct value of the corresponding vehicle control.

**Figure 3:**
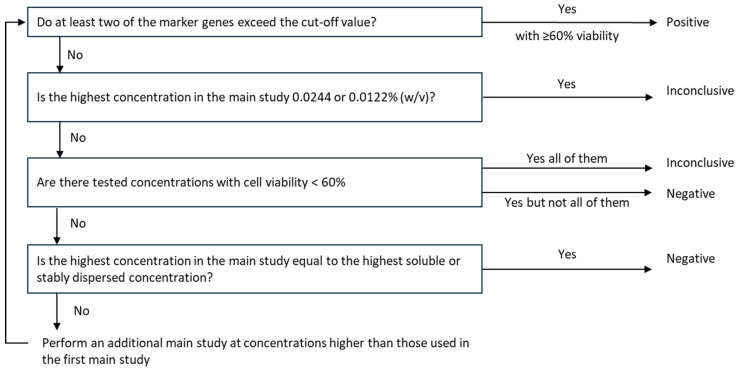
Flow-chart of the Epi2SensA prediction model.

Individual genes were considered activated when the fold induction exceeded their respective cut-off values: ATF3 > 15, GCLM > 2, DNAJB4 > 2, IL8 > 4. The prediction model of the VRM initially required that at least one of the genes exceed the established cut-off value to be classified as a sensitizer. This rule was modified to a minimum two genes being activated for classification of a test substance as a sensitizer in the Epi2SensA protocol (Figure 3).

### 2.8. Statistical Analysis

Mattek conducted 12 runs for testing the 44 coded samples, of which one was invalid. Eurofins and BRT submitted 13 runs each, including 1 and 2 invalid runs, respectively. FDSC conducted two runs, both valid. The invalidity was due to positive control responses below the thresholds, and a new test was performed each time (see Supplementary Table S2). Within-laboratory reproducibility (WLR) was calculated based on concordance of predictions for 12 chemicals tested by each laboratory in three independent runs. Between-laboratory reproducibility (BLR) was assessed using concordance of predictions across the four laboratories for the 20 reference chemicals. For substances tested in three runs for WLR assessment, the mode of the three predictions was used (i.e., the most frequently occurring result from the three runs) for BLR calculations. Predictive capacity was determined by comparing Epi2SensA predictions to in vivo reference classifications. For repeatedly tested substances, the mode of the three predictions was used. Sensitivity, specificity, accuracy, and balanced accuracy were calculated.

## 3. Results

### 3.1. Adaptation of the SOP to the EpiDerm™ Model

**Figure 4:**
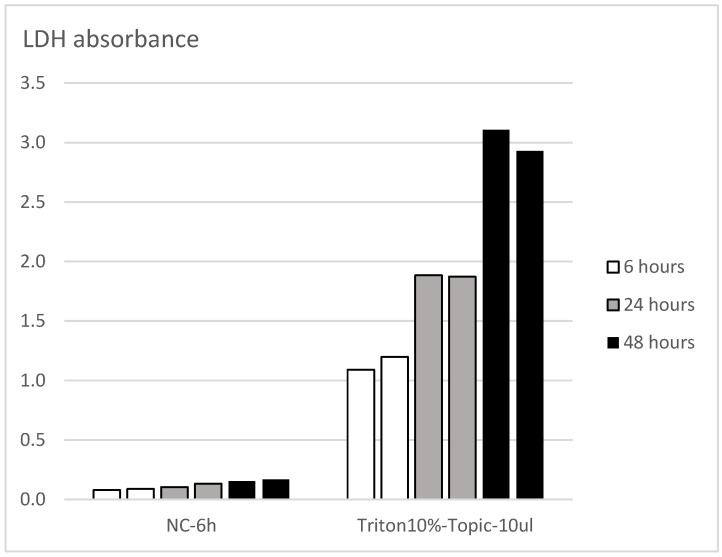
Kinetics of LDH release after exposure of the EpiDerm model to 10 μL of PBS (NC) and to 10 μL of 10% Triton-100 (individual data from two tissue replicates per condition are presented).

**Figure 5:**
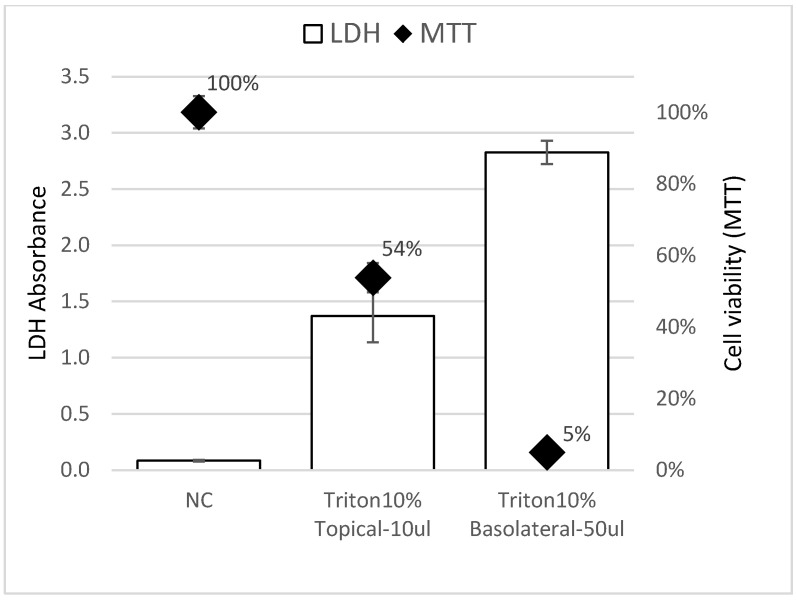
Comparison of LDH amount (bar, left vertical axis) measured in the medium with the percentage of cell viability measured by the MTT (diamond, right vertical axis) method. EpiDerm™ tissues were exposed during 6 h topically to 10 μL of a 10% Triton-X-100 solution or to 50 μL of 10% Triton-X-100 added to 1.0 mL of medium in the basolateral compartment. NC (negative control) tissues were exposed to DPBS (data are presented as mean of 3 replicates from 1 experiment; error bars represent standard deviation of the replicates).

The volume of test article applied to the tissues was scaled to the surface area of the EpiDerm model. The volume was increased from 5 μL in the VRM to 10 μL for Epi2SensA to maintain the same volume/surface area ratio as in the EpiSensA assay. The exposure duration was shortened to 1 h to reduce unexpected cytotoxicity observed with the EpiDerm™ model, while maintaining the 6 h time point for gene expression. In addition, the procedure for killed controls was adapted since it was recognized that the procedure used in the VRM to lyse the tissues (used to establish the 100% LDH release reference value) was not effective in fully releasing LDH from the EpiDerm™ model (Figure 4). This would lead to overestimates of cytotoxicity. Using the VRM procedure for killed control tissues and EpiDerm tissues, 54% residual cell viability was measured with the MTT method (Figure 5). Therefore, the procedure for the killed controls was modified—50 μL of 10% Triton-X-100 was added to 1 mL of the medium in the basolateral compartment (Table 2). Under these conditions, residual viability as measured by the MTT assay was only 5% (Figure 5). Experimental data showed this modification to be effective in releasing essentially all the tissue’s LDH so that the correct cell viability would be calculated.

Finally, the concentration of the positive control, clotrimazole, was increased from 0.78% to 1.56% in order to reduce the risk of run failure that had occasionally been observed at the lower concentration during development at Mattek (Table 2).

### 3.2. Similar Method Validation Study

After checking runs for validity (see Supplementary Table S2), analyses performed by the biostatistician were conducted by applying the acceptance criteria of the performance standards (PS) [11]. The calculated average WLR was 63.9%, and the BLR was 70%, both below the 80% specified in the PS. The average predictive capacity was 66.6% specificity and 90.5% sensitivity, exceeding the minimum PS values of 65% and 85%, respectively, and an accuracy of 83.3%, which was close to the minimum PS value of 85%. Results were re-analyzed using the modified acceptance criteria for the Epi2SensA method by using a lower tissue viability cut-off (≥60%) after analysis of the initial performance data. Observing a few positive predictions that were based on the induction of a single gene only, the core VMT agreed to modify the prediction model by requiring at least two genes exceeding the gene-specific induction threshold to support a positive prediction. Incorporating these modifications into the prediction model resulted in increased reproducibility and increased average WLR for the three laboratories of 83.3% and the BLR of 85%, meeting both the acceptance criteria. The predictive capacity using the modified acceptance criteria was then determined by comparing the Epi2SensA predictions with the in vivo predictions specified in the PS. The average predictive capacity was 88.9% specificity, 88.1% sensitivity, and 88.3% accuracy, which exceeded the minimum PS values of 65%, 85%, and 85%, respectively (Table 3).

### 3.3. Applicability of the Test Method

The Epi2SensA and the EpiSensA methods are based on the use of RhE models to assess induction of four genes related to keratinocyte activation (KE2) during the initial responses leading to allergenic reactions. The EpiDerm^TM^ model and the LabCyte EPI-MODEL 24, used in the Epi2SensA and EpiSensA methods, respectively, have been validated for skin corrosion (OECD TG431) and skin irritation (OECD TG439), where they share the same applicability domain.

Since RhE models allow the application of test substances directly to the surface of the tissue, the exposure conditions are similar to those of in vivo tests. Products in a lipophilic vehicle can be tested, overcoming the limitation for hydrophobic compounds in submerged culture models [13,14]. Table 4 presents the performance of Epi2SensA for the validation set including the lipophilic chemicals (e.g., LogP ≥ 3.5). The RhE models are highly differentiated skin epithelia with metabolic capacity and therefore substances that require metabolism activation can also be tested [15,16]. Performance of Epi2SensA in testing known pre/pro-haptens is shown in Table 4. Epi2SensA correctly classified 5 of 6 pre/pro-haptens that were included in the validation set of substances.

Of the 20 test chemicals in the validation set, 2 were classified as false positives by the VRM (Diethyl phthalate and 1 Iodohexane) and 1 as a false negative (Lauryl Gallate); 6 are pre/pro-haptens and 9 are hydrophobic/lipophilic substances with a LogP ≥ 3.5 (Table 4). Looking more specifically at the substances that were sources of variability across laboratories, it should be noted that these substances had already been identified as extremely challenging in the VRM, leading to the conclusion for Benzisothiazolinone that “it may be difficult to obtain accurate results consistently, with a strong likelihood of non-concordant outcomes across laboratories” and for Cetrimide that it “is known to be relatively difficult to identify as a non-sensitizer” [17].

## 4. Discussion

Integration of methods using RhE models into testing strategies for the assessment of chemical sensitization offers several advantages. As demonstrated with validated methods for irritation, corrosion, or photo-irritation, RhE models allow testing under conditions that mimic clinical exposure through direct topical application of water soluble and non-water-soluble test materials. Moreover, the presence of a functional stratum corneum enables testing without physicochemical restrictions on liquids, solids, water-soluble or liposoluble substances, as well as complex mixtures. Compared to in chemico systems or cell lines generally used in in vitro tests, RhE models possess xenobiotic metabolic activity, which allows the identification of pro-haptens requiring metabolic activation to become sensitizers [7]. In 2024, the OECD TG442D integrated the first method using an RhE model for skin sensitization assessment, the EpiSensA method, using the LabCyte RhE model. Since the EpiDerm™ model has long been used in several OECD guidelines (OECD TG431, 439 and 498) and has worldwide availability, it was a natural choice to engage a similar method validation of the Epi2SensA method.

The Epi2SensA assay quantifies the expression of four mechanistically anchored genes, ATF3, IL-8, GCLM, and DNAJB4, that have been previously established as biomarkers of keratinocyte activation by skin sensitizers in the validated reference method (EpiSensA) [10]. Each of these genes has been demonstrated to be associated with a distinct biological pathway that is pertinent to KE2 of the skin sensitization AOP. ATF3 is a central regulator of the cellular adaptive response, modulating cytokine and chemokine expression through ATP- and NF-κB-dependent signaling [18,19]. IL-8 is a pro-inflammatory cytokine that functions as a chemotactic mediator for neutrophils. Its expression is regulated by ATP–P2 × 7 receptor activation and downstream signaling through the p38 MAPK pathway [7,20]. GCLM encodes the modifier subunit of glutamate-cysteine ligase, the rate-limiting enzyme for glutathione biosynthesis. Its expression is regulated by the nuclear factor E2-related factor 2 (Nrf2)/antioxidant response element (ARE) and activator protein-1 (AP-1) pathways, thus linking oxidative stress to cytoprotective responses [7,17]. DNAJB4, a heat shock protein co-chaperone involved in stabilizing protein folding under oxidative stress [21], is regulated by the Nrf2/ARE and the heat shock transcription factor-1/heat shock factor response element (HSF-1/HSE) pathways [22,23]. Collectively, these markers signify cytoprotective, antioxidant, and inflammatory processes that are characteristic of keratinocyte activation upon sensitizer exposure.

Compared to the VRM, the Epi2SensA protocol incorporates tissue-specific modifications for the EpiDerm™ model: increased applied volume, reduced exposure duration, and a modified procedure to generate killed controls (Table 2). To evaluate the similarity of Epi2SensA to the VRM, the method was assessed in a validation study according to the ‘Performance Standards for the Assessment of Proposed Similar or Modified In Vitro Epidermal Sensitisation Assay (EpiSensA) Test Methods’ [11]. The assay was transferred by the lead laboratory to three naïve laboratories, demonstrating method feasibility for implementation across different organizations. The requirement for technical troubleshooting and protocol refinement during laboratory onboarding, particularly regarding RNA extraction methodology and RT-qPCR optimization, underscored the importance of comprehensive training, documentation and ongoing technical support for method implementation.

The experimental phase of the validation study was conducted from September 2024 to August 2025, after which the results from 40 runs were submitted to an independent biostatistician for statistical analysis. Initial analyses used the acceptance criteria and decision algorithm outlined in the VRM and showed excellent predictivity, with an average sensitivity of 90.5%, specificity of 66.6%, and accuracy of 83.3%. However, the average WLR of 63.9% and the BLR of 70% reflected a variability in the results that did not meet the performance criteria of the VRM.

Subsequent statistical analyses incorporated two modifications specific to the EpiDerm™ tissue model, namely, the reduction in the acceptable cytotoxicity threshold from 80% to 60% and a positive prediction now requiring the induction of at least two genes above their respective thresholds. Under these conditions, the WLR was 83.3% and the BLR was 85.0%. The method achieved a sensitivity of 88.0%, an overall accuracy of 88.3%, and a specificity of 88.9%, meeting all performance criteria.

The implementation of tissue-specific acceptance criteria for Epi2SensA led to a substantial improvement in method performance. For tissue viability, preliminary studies by the lead laboratory showed the tendency for the LDH method to overestimate cytotoxicity with the EpiDerm™ model [Figure 4 and Figure 5]. Although this issue has been addressed by modifying the method used to obtain killed controls, it cannot be completely excluded that this tendency persists. More broadly, validated OECD methods for skin irritation use 50% as a cut-off value for a non-irritant chemical [24]. It is also worth noting that EpiSensA was originally developed using the EpiDerm™ model, with an acceptance criterion for tissue viability set at 50% [9]. This was later raised to 80% when the method was adapted to the Labcyte model [10]. The 60% threshold of cell viability implemented in Epi2SensA allows detection of sensitization responses that occur at concentrations causing mild/moderate cytotoxicity. Many toxic and stress pathways (DNA damage, oxidative stress, apoptosis, inflammation) are robustly activated when cells are put under sufficient stress, which typically happens at concentrations that start to reduce viability [25,26]. Using a cell viability acceptance of 60% includes tested concentrations where the cellular stress response is clearly engaged, increasing fold induction of biomarker genes and improving signal-to-noise ratio.

The second major refinement in the Epi2Sensa method involves the prediction algorithm, which now requires at least two of the four marker genes (ATF3, IL-8, GCLM, DNAJB4) to be induced over their respective induction thresholds to support a positive classification. This modification was driven by the fact that these genes capture both pro-inflammatory and cytoprotective pathways associated with KE2. Requiring activation of at least two markers increases the mechanistic coherence of a positive classification and reduces the likelihood that an isolated signal may reflect non-specific stress or experimental variability. In practice, this adjustment significantly decreased the risk of false positive classifications, resulting in substantially improved method specificity (Table 3). OECD Guidance Document No. 34 explicitly recognizes that “at the completion of a validation study, there may be situations where the data clearly indicate that the decision criteria need to be refined in order to increase the predictive capacity.” This provision specifically allows for data-driven modifications to decision criteria based on actual validation results, particularly for “me-too” methods using different tissue models [27]. The maintenance of the predictive performance after these adaptations of the Epi2SensA model was confirmed after the validation study on an extended panel of 47 chemicals (manuscript in preparation).

## 5. Conclusions

The Epi2SensA method, based on the EpiDerm™ model, met the acceptance criteria for validation of a similar method to the validated reference method (VRM). Specific assay acceptance criteria for the EpiDerm model, a 60% viability threshold and a two-gene induction algorithm, substantially enhanced method performance without compromising the scientific validity of the method. Successful transfer to three naïve labs confirmed multi-site feasibility with proper training and support. Interlaboratory validation across four labs and 20 reference chemicals yielded strong results: 85% between-laboratory reproducibility (exceeding the ≥80% threshold) and 83.3% average within-laboratory reproducibility (meeting the ≥80% requirement). Predictive performance was robust, with 88.1% sensitivity, 88.9% specificity, and 88.3%, accuracy, all surpassing performance criteria (≥85% sensitivity/accuracy, ≥65% specificity).

The demonstrated performance using the EpiDerm™ model supports regulatory acceptance of Epi2SensA as a validated method. Epi2SensA integration in OECD TG442D will provide laboratories with multiple RhE supplier options and ensure worldwide supply chain reliability.

## Figures and Tables

**Table 1:** Minimum list of reference substances for determination of reproducibility and predictive capacity of similar or modified EpiSensA test methods (adapted from EpiSensA’s PS) [11].

No.	Proficiency Substances	CAS No.	Physical State	In Vivo Prediction ^1^	LogP	Pre/Pro-Hapten	Vehicle ^2^	VRM Prediction
1	2,4-Dinitrochlorobenzene	97-00-7	Solid	Sensitizer (GHS Cat.1A)	2.17		AOO	Positive
2	p-Phenylenediamine	106-50-3	Solid	Sensitizer (GHS Cat.1A)	−0.3	X	AOO	Positive
3	Metol	55-55-0	Solid	Sensitizer (GHS Cat.1A)	0.63	X	DW	Positive
4	Tetrachloro-salicylanilide	1154-59-2	Solid	Sensitizer (GHS Cat.1A)	5.87		AOO	Positive
5	Lauryl galate	1166-52-5	Solid	Sensitizer (GHS Cat.1A)	6.9	X	AOO	Negative
6	Methyl heptine carbonate	111-12-6	Liquid	Sensitizer (GHS Cat.1A)	2.79		AOO	Positive
7	Isoeugenol	97-54-1	Liquid	Sensitizer (GHS Cat.1A)	3.04	X	AOO	Positive
8	Glyoxal 40% solution in water	107-22-2	Liquid	Sensitizer (GHS Cat. 1A	−0.08		DW	Positive
9	Abietic acid	514-10-3	Solid	Sensitizer (GHS Cat.1B)	3.92	X	AOO	Positive
10	Dibutyl aniline	613-29-6	Liquid	Sensitizer (GHS Cat.1B)	4.7	X	AOO	Positive
11	Amyl cinnamic aldehyde	122-40-7	Liquid	Sensitizer (GHS Cat.1B)	3.99		AOO	Positive
12	Benzisothiazolinone	2634-33-5	Solid	Sensitizer (GHS Cat.1B)	0.8		AOO	Positive ^4^
13	Imidazolidinyl urea	39236-46-9	Solid	Sensitizer (GHS Cat.1B)	−0.86		DW	Positive
14	Farnesol	4602-84-0	Liquid	Sensitizer (GHS Cat.1B)	4.91		AOO	Positive
15	Cetrimide	57-09-0	Solid	Non-sensitizer (Not classified)	3.18		50% EtOH	Negative
16	Lactic acid ^3^	50-21-5	Liquid	Non-sensitizer (Not classified)	−0.72		DW	Negative
17	Benzyl butyl phthalate	85-68-7	Liquid	Non-sensitizer (Not classified)	4.84		AOO	Negative
18	Diethyl phthalate	84-66-2	Liquid	Non-sensitizer (Not classified)	2.44		AOO	Positive
19	Hexane	110-54-3	Liquid	Non-sensitizer (Not classified)	3.9		AOO	Negative
20	1-Iodehexane	638-45-9	Liquid	Non-sensitizer (Not classified)	3.99		AOO	Positive

^1^: The in vivo hazard and potency prediction is based on LLNA data [5]. The in vivo potency is derived using the criteria based on UN GHS Sub-categorization. ^2^: Based on historical results [10,11]. ^3^: MTT assay should be performed instead of LDH assay. ^4^: Reference substance which was not 100% concordant between laboratories. Gray shading: A subset of 12 out of the 20 reference substances that should be used for an assessment of within-laboratory reproducibility (WLR).

**Table 2:** Comparison of Epi2SensA with the essential test method components and performance of the VRM (EpiSensA).

Essential Test Method Component	VRM (EpiSensA)	Epi2SensA	Key Difference and Rationale
Reconstructed Human Epidermis (RhE) Model	LabCyte EPI-MODEL24.	EpiDerm (EPI-200).	Different model. This was the fundamental difference requiring subsequent protocol adjustments.
Marker Genes	Quantifies expression of ATF3, GCLM, DNAJB4, and IL-8.	Quantifies expression of ATF3, GCLM, DNAJB4, and IL-8.	Identical. Both methods target the same four mechanistically relevant genes associated with keratinocyte activation.
Gene Cut-off Values	ATF3 > 15-fold; GCLM > 2-fold; DNAJB4 > 2-fold; IL-8 > 4-fold.	ATF3 > 15-fold; GCLM > 2-fold; DNAJB4 > 2-fold; IL-8 > 4-fold.	Identical. The gene-specific induction thresholds were retained.
Cytotoxicity Viability Threshold	Must maintain cell viability > 80% for acceptable test concentration results.	Must maintain cell viability > 60% for acceptable test concentration results.	Modified criterion. The threshold was reduced from 80% to 60% based on preliminary data showing the LDH assay overestimated cytotoxicity for EpiDerm compared to the MTT assay and to enhance test reproducibility.
Prediction Model (Positive Result)	Prediction is positive if at least one marker gene exceeds its cut-off (Imax) at an accepted concentration.	Prediction is positive if at least two marker genes exceed their respective cut-off values (Imax) at an accepted concentration.	Modified criterion. The requirement was increased to two positive genes to enhance robustness, a modification common when using different tissue models in similar method validation.
Exposure Time	6 h.	1 h topical exposure followed by a 5 h post-incubation period.	Modified procedure. The exposure duration was shortened to 1 h to reduce unexpected cytotoxicity observed with the EpiDerm model, while maintaining the 6 h time point for gene expression measurement.
Application Volume	5 μL applied to the epidermis surface.	10 μL applied to the epidermis surface.	Modified procedure. The volume was doubled because the surface area of the EpiDerm model (0.63 cm2 is roughly double that of the LabCyte model (0.32 cm2) thus maintaining a similar volume/surface ratio.
Positive Control (Clotrimazole)	0.78% (*w*/*v*).	1.56% (*w*/*v*).	Modified procedure. The concentration was increased to ensure the run acceptance criteria were consistently met for ATF3 and IL-8 fold induction, as 0.78% led to a 40% failure rate in preliminary Epi2SensA runs.
Killed Control Method	10 μL of 10% Triton X-100 applied topically.	50 μL of 10% Triton X-100 applied in the culture medium.	Modified procedure. Changed the volume and application to ensure complete tissue death with maximum LDH release for the EpiDerm model.

**Table 3:** Comparison of WLR, BLR and predictive capacity to performance standard acceptance criteria using the original VRM criteria and the modified Epi2SensA criteria.

	Acceptance Criterion	Average Original VRM	Average Modified Epi2SensA	Mattek		Eurofins		BRT	
				Original VRM	Modified Epi2SensA	Original VRM	Modified Epi2SensA	Original VRM	Modified Epi2SensA
**WLR**	**≥80%**	63.9%	**83.3%**	66.7% (8/12)	**91.7%** (11/12)	58.3% (8/12)	**83.3%** (10/12)	66.7% (8/12)	**75.0%** (9/12)
**BLR**	**≥80%**	70%	**85%**	Original VRM: 70%			Modified Epi2SensA: **85%**		
**Specificity**	**≥65%**	66.6%	**88.9%**	66.7% (4/6)	**100%** (6/6)	83.3% (5/6)	**83.3%** (5/6)	50.0% (3/6)	**83.3%** (5/6)
**Sensitivity**	**≥85%**	90.5%	**88.1%**	92.9% (13/14)	**92.9%** (13/14)	85.7% (12/14)	**78.6%** (11/14)	92.9% (13/14)	**92.9%** (13/14)
**Accuracy**	**≥85%**	83.3%	**88.3%**	85.0% (17/20)	**95.0%** (18/20)	85.0% (17/20)	**80.0%** (16/20)	80.0% (16/20)	**90.0%** (18/20)

**Table 4:** Performance of Epi2SensA for the 20 chemicals of the validation set (PF).

Chemical	CAS	Log P	Pre/Pro-Hapten	Classification UN GHS In Vivo	VRM Classification	Epi2SensA Classification
2,4-Dinitrochlorobenzene	97-00-7	2.17		UN GHS Cat. 1A	Sensitizer	Sensitizer
p-Phenylenediamine	106-50-3	−0.39	Pre	UN GHS Cat. 1A	Sensitizer	Sensitizer
Metol	55-55-0	0.63	Pre/Pro	UN GHS Cat. 1A	Sensitizer	Sensitizer
Tetrachlorosalicylanilide	1154-59-2	5.87		UN GHS Cat. 1A	Sensitizer	Sensitizer
Lauryl gallate	1166-52-5	6.9	Pre	UN GHS Cat. 1A	Non-sensitizer	Non-sensitizer
Methyl heptine carbonate	111-12-6	2.79		UN GHS Cat. 1A	Sensitizer	Sensitizer
Isoeugenol	97-54-1	3.04	Pre/Pro	UN GHS Cat. 1B	Sensitizer	Sensitizer
Glyoxal	107-22-2	−0.08		UN GHS Cat. 1A	Sensitizer	Sensitizer
Abietic acid	514-10-3	3.92	Pre	UN GHS Cat. 1B	Sensitizer	Sensitizer
Dibutyl aniline	613-29-6	4.7	Pro	UN GHS Cat. 1B	Sensitizer	Sensitizer
Amyl cinnamic aldehyde	122-40-7	3.99		UN GHS Cat. 1B	Sensitizer	Sensitizer
Benzisothiazolinone	2634-33-5	0.8		UN GHS Cat. 1B	Sensitizer	Sensitizer
Imidazolidinyl urea	39236-46-9	−0.86		UN GHS Cat. 1B	Sensitizer	Sensitizer
Farnesol	4602-84-0	4.91		UN GHS Cat. 1B	Sensitizer	Sensitizer
Cetrimide	57-09-0	3.18		Not classified	Non-sensitizer	Non-sensitizer
Lactic acid	50-21-5	−0.72		Not classified	Non-sensitizer	Non-sensitizer
Benzyl butyl phthalate	85-68-7	4.84		Not classified	Non-sensitizer	Non-sensitizer
Diethyl phthalate	84-66-2	2.44		Not classified	Sensitizer	Non-sensitizer
Hexane	110-54-3	3.9		Not classified	Non-sensitizer	Non-sensitizer
1-Iodehexane	638-45-9	3.99		Not classified	Sensitizer	Sensitizer

## Data Availability

Data is contained within the article and its Supplementary Materials.

## Supplementary Materials

The following supporting information can be downloaded at: https://www.mdpi.com/article/10.3390/toxics14040295/s1, Table S1: Summary data; Table S2: Overview of run validity;

## Abbreviations

The following abbreviations are used in this manuscript:

2D	2-dimensional
4NBB	4-Nitrobenzyl bromide
AOO	acetone:olive oil (20% v/v olive oil in acetone)
AOP	adverse outcome pathway
AP-1	activator protein-1
ATF3	Activating Transcription Factor 3
BLR	between-laboratory reproducibility
BRT	Burleson Research Technologies, Inc.
Ct	Cycle threshold
DA	Defined Approach
DNAJB4	DnaJ homolog subfamily B member 4
DPBS	Dulbecco’s Phosphate-Buffered Saline
DW	distilled water
EtOH	ethanol
FDSC	Food and Drug Safety Center
GAPDH	Glyceraldehyde-3-phosphate dehydrogenase
GARD	Genome Allergen Rapid Detection
GCLM	Glutamate-Cysteine Ligase Modifier subunit
h-CLAT	human cell line activation test
HSF-1/HSE	heat shock transcription factor-1/heat shock factor response element
IATA	Integrated Approaches to Testing and Assessment
IL-8	Interleuikin-8
IL-8 Luc	Interleukin-8 Reporter Gene Assay
ITS	Integrated Testing Strategy
KE	key event
LDH	lactate dehydrogenase
LLNA	Local Lymph Node Assay
LogP	logarithm of the Octanol-Water Partition Coefficient
MTT	3-[4,5-dimethylthiazol-2-yl]-2,5 diphenyl tetrazolium bromide
Nrf2/ARE	nuclear factor E2-related factor 2/antioxidant response element
OECD	Organisation for Economic Co-operation and Development
PS	Performance Standard
RhE	reconstructed human epidermis
RNA	ribonucleic acid
RT-qPCR	quantitative real-time polymerase chain reaction
SOP	Standard Operating Procedure
TG	test guideline
U-SENS™	U937 Cell Line Activation Test
VMT	Validation Management Team
VRM	Validated Reference Method
WLR	within-laboratory reproducibility
Δabs	change in absorbance
